# Hereditary Hemochromatosis Associated With Idiopathic Refractory Aplastic Anemia in a Five-Year-Old Boy: A Case Report

**DOI:** 10.7759/cureus.20135

**Published:** 2021-12-03

**Authors:** Ibrahim Alharbi, Abdullah K Bahakim, Sanad M Alharthi, Saad M Alharthi, Abdulrahman A Baabdullah

**Affiliations:** 1 Pediatric Hematology Oncology, King Fahad Armed Forces Hospital, Jeddah, SAU; 2 Department of Pediatrics, Umm Al-Qura University, Makkah, SAU; 3 Medicine and Surgery, Umm Al-Qura University, Makkah, SAU

**Keywords:** bone marrow failure, pancytopenia, iron overload, aplastic anemia, hereditary hemochromatosis

## Abstract

Hereditary hemochromatosis (HH) is a multisystem disease characterized by iron overload and various clinical presentations, including cirrhosis, diabetes mellitus, and heart failure. HH can be caused by the human homeostatic iron regulator (HFE) and non-HFE gene mutations. Aplastic anemia is a rare, life-threatening bone marrow failure in which fat replaces pluripotent stem cells, resulting in pancytopenia and hypoplasia of bone marrow. We present a case of a five-year-old-boy who initially presented with a large ecchymosis located at the right side of the chest and abdomen. These started suddenly after minor trauma. Later, he was diagnosed with idiopathic aplastic anemia and treated with immunosuppressive therapy (IST). As part of the workup for pancytopenia, we ordered whole exome sequencing (WES) and diagnosed the patient with autosomal recessive hereditary hemochromatosis (ARHH). The ARHH is caused by HFE pathogenic gene mutation variant (c.187C>G p homozygous genotype). After six months of IST, he still had persistent disease. Human leukocyte antigen (HLA) typing showed he has a sister who is a full match but also has ARHH. Because of this, a haploidentical hematopoietic stem cell transplantation (hHSCT) from the father was performed. The hHSCT had a successful outcome. We suggest that in children with idiopathic aplastic anemia, physicians should be aware of the possibility of co-existing hereditary hemochromatosis or secondary hemochromatosis. Serum ferritin and transferrin saturation should also be measured regularly in order to detect early hemochromatosis.

## Introduction

Hereditary hemochromatosis (HH) is a multisystem disease characterized by enhanced iron absorption in the intestines, leading to excess iron deposition in many organs (such as the liver, heart, pancreas, joints, skin, and endocrine glands that disturb their functions) and presenting with complications (such as cirrhosis, heart failure, diabetes mellitus, arthralgias, skin hyperpigmentation, and hypogonadism). HH is the most commonly identified genetic disorder amongst Caucasians [1].

Primary (hereditary) hemochromatosis is an autosomal recessive disorder caused more frequently by human homeostatic iron regulator (HFE) gene mutation, whose protein product control iron absorption from the intestines and is less frequently caused by other non-HFE gene mutations [2]. Secondary hemochromatosis is developed when a buildup of iron stems from other medical conditions, such as repeated blood transfusion, ineffective erythropoiesis, and excessive alcohol consumption [1].

Laboratory tests have a remarkable contribution to the diagnosis of HH as almost all cases show elevated serum ferritin and transferrin iron saturation of more than 45%. Genetic assay for C282Y, H63D homozygous genotype known as HFE gene mutation is the next step for diagnosing HH. Therapeutic phlebotomy is the mainstay of treatment for HH [3].

Aplastic anemia (AA) is a rare, life-threatening bone marrow failure in which fat replaces pluripotent stem cells, resulting in pancytopenia and hypoplasia of bone marrow. Although the condition is rare in children, when it is seen, the peak age of incidence is between three and five years [4]. Most cases of AA are idiopathic and have unexplained etiologies. Secondary AA is usually related to drugs, exposure to toxic substances, radiation, viral infections, and rheumatic diseases [5]. The treatment for AA is initiated based on age, clinical status, and severity of the disease. It can range from observation, blood transfusions, immunosuppressants, and bone marrow transplantation [6]. This paper reports a unique case of a five-year-old boy presented as idiopathic AA and later diagnosed with HH by ordering the genetic tests. We propose that physicians should be aware of the likelihood of co-existence of HH in children with AA.

## Case presentation

A five-year-old Saudi boy without a relevant medical or family history presented with a three-week history of small petechiae that started in the face and then spread to other parts of his body, without seeking any medical advice. On the presentation day, the patient’s parents brought their child to the emergency department (ED) with a large ecchymosis that was present for two days. It was located at the right side of his chest down to the right side of his abdomen. It started suddenly after minor trauma. The patient had no history of a previous similar event. Also, there was no history of epistaxis, hematemesis, or gum bleeding. There were no associated symptoms, such as abdominal pain, bone pain, urine color change, or changes in stool color/melena/hematochezia. The systemic review was unremarkable. He had no fevers, weight loss, loss of appetite, fatigue, or night sweating. The patient did not use any medications, receive blood transfusions, or have a family history of bleeding disorders. On physical examination, the patient was pale and had stable vital signs, with unremarkable findings except for ecchymosis at the right side of the chest and abdomen as well as petechiae all over the body. No palpable lymphadenopathy in any sites or hepatosplenomegaly was identified. The initial complete blood count (CBC) revealed pancytopenia (Table 1). Renal and liver function tests and coagulation profiles were all normal (Table 1). Neonatal direct Coomb’s test was negative. G6PD screening and quantitative tests were normal levels. The viral serology and autoimmune profile were negative, which excluded viral infections and autoimmune diseases, respectively (Table 2).

**Table 1:** Laboratory investigations at the time of admission. WBC: white blood cell, RBC: red blood cell, MCV: mean corpuscular volume, MCH: mean corpuscular hemoglobin concentration, ALT: alanine transaminase, AST: aspartate transaminase, ALP: alkaline phosphatase, PT: prothrombin time, PTT: partial thromboplastin time, INR: international normalized ratio, BUN: blood urea nitrogen. *WBC normal range: (5.5–10 K/μL)

Complete blood count	Values	Unit
WBC	5.32*	K/μL
Neutrophils	9.4	%
Lymphocytes	86.3	%
RBC	2.70	M/μL
Hemoglobin	8.2	g/dL
Hematocrit	22.0	%
MCV	81.5	fL
MCH	30.4	pg
Platelets	2	K/μL
Reticulocyte absolute	0.87	%
Blood chemistry		
Albumin	43	g/L
ALT	12	U/L
AST	16	U/L
ALP	221	U/L
Total bilirubin	14.6	mg/dL
Direct bilirubin	8.8	mg/dL
PT	12.1	s
PTT	26.1	s
INR	1.0	
BUN	4.0	mg/dL
Creatinine, serum	29.0	
Ferritin levels	1,947	mcg/dL
Transferrin saturation	312	mcg/dL
Iron	35	umol/L
Total iron-binding capacity	44.5	umol/L
Transferrin saturation	285	mcg/dL
Vitamin B12 level	151	pmol/L
Folate level	2,280	nmol/L

**Table 2:** Autoimmune and viral serology profiles. ANA: antinuclear antibody, AMA: antimitochondrial antibody, C-ANCA: cytoplasmic antineutrophil cytoplasmic antibodies, P-ANCA: perinuclear antineutrophil cytoplasmic antibody, SMA: smooth muscle antibody, CMV IgG: cytomegalovirus immunoglobulin G, EBV IgM: Epstein Barr virus immunoglobulin M, HCV: hepatitis C, HAV: hepatitis A, HBsAg: hepatitis surface antigen, HIV: human immunodeficiency virus, VDRL: venereal disease research laboratory.

Test name	Result
ANA	Negative
AMA	Negative
C‐ANCA	Negative
P‐ANCA	Negative
SMA	Negative
CMV IgG	Negative
EBV IgM	Negative
HCV antibodies	Negative
HAV IgM	Negative
HBsAg	Negative
HIV	Negative
VDRL	Negative

**Figure 1:**
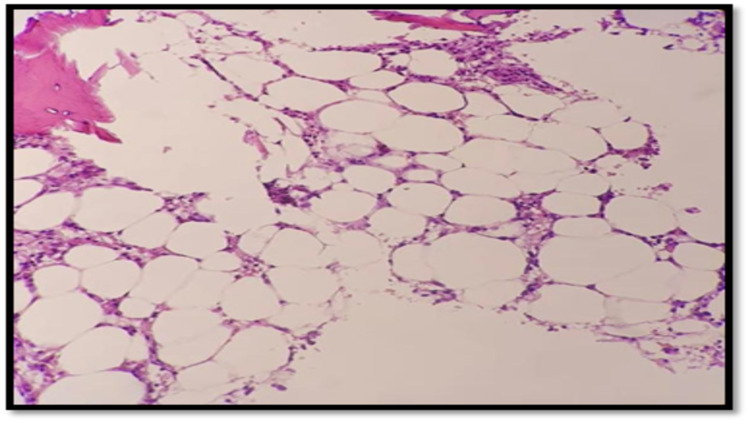
Bone marrow biopsy indicated marked hypocellular bone marrow with reduced cellularity.

**Figure 2:**
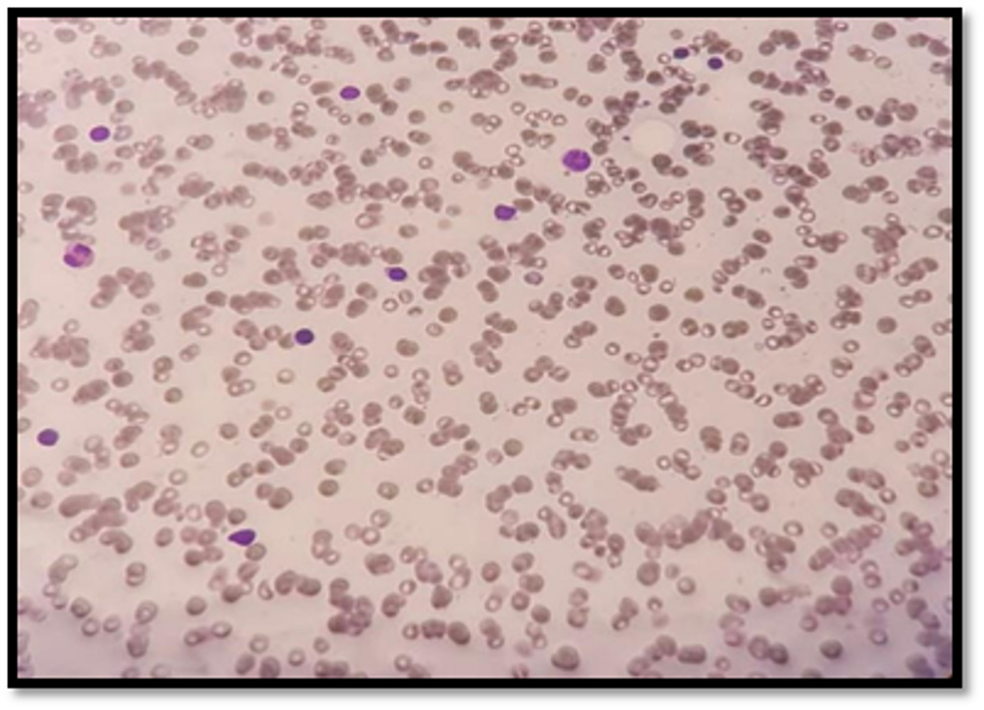
Bone marrow aspiration showed markedly hypocellular marrow with a reduction of all elements.

Based on the findings mentioned above, aplastic anemia was suspected. The patient was planned for bone marrow aspiration and biopsy (BMA and BMB) to establish the diagnosis. Bone marrow aspirate and biopsy showed markedly hypocellular bone marrow with cellularity (10-20%) (Figure 1). Bone marrow aspiration showed markedly hypocellular marrow with reduction of all elements; however, neither abnormal cells nor dysplastic features were seen (Figure 2). This finding is consistent with aplastic anemia. The immunophenotype report showed no evidence of B-cell monoclonality, acute leukemia, or T-cell lymphoproliferative disorder. Eventually, the patient was diagnosed with idiopathic aplastic anemia. Genetic panel testing was requested (sequencing including next-generation sequencing-based copy number variation analysis), and it revealed a homozygous pathogenic variant (c.187C>G p) was identified in the HFE gene. This finding is consistent with the genetic diagnosis of autosomal recessive hemochromatosis type 1.

The patient received multiple platelets and packed red blood cells (PRBCs) transfusions during his hospital visit. The patient was started on immunosuppressive therapy (IST) that consisted of anti-thymocyte globulin (ATG), prednisolone, cyclosporine (CSA), and eltrombopag (thrombopoietin receptor against).

During this period, human leukocyte antigen (HLA) typing was performed for the patient and his sister for bone marrow transplantation. He had a matched HLA sister donor for a bone marrow transplant. Unfortunately, his sister has the same homozygous pathogenic variant that was identified in her brother. There was a conflict in decision-making among the doctors, which lead to not performing a bone marrow transplantation for the patient’s safety. HLA typing of the patient and his father showed half HLA compatibility. Ultimately, the transplantation has carried out nine months after his initial presentation.

The patient was initially discharged from the hospital on CSA and prednisolone. During follow‐up in the clinic, his CBC, renal, and liver function test results were checked frequently, and his CSA level was monitored.

Within the following months, the patient had repeated visits to the emergency department (ED), presenting with symptoms related to anemia (such as fatigue), or symptom related to thrombocytopenia (such as gum bleeding, for which he received blood transfusions) or symptoms related to leukocytopenia (such as fever with persistent watery diarrhea). His condition remained like this, with no response to the immunosuppressive treatment approximately six months after the initial presentation. Then the patient arranged for bone marrow transplantation. One month after the transplantation, the patient showed marked improvement in his condition and his symptoms are resolved and to the time of writing this manuscript, the patient completed five months with no active complaint. The last CBC report is shown in (Table 3).

**Table 3:** Laboratory investigations after bone marrow transplant. WBC: white blood cell, RBC: red blood cell, MCV: mean corpuscular volume, MCH: mean corpuscular hemoglobin concentration, ALT: alanine transaminase, AST: aspartate transaminase, ALP: alkaline phosphatase, PT: prothrombin time, PTT: partial thromboplastin time, INR: international normalized ratio, BUN: blood urea nitrogen.

Complete blood count	Values	Unit
WBC	6.21	K/μL
Neutrophils	61.3	%
Lymphocytes	20.8	%
RBC	2.98	M/μL
Hemoglobin	9.3	g/dL
Hematocrit	27.2	%
MCV	91.3	fL
MCH	28.5	pg
Platelets	247	K/μL
Reticulocyte absolute	1.80	%
Blood chemistry		
Albumin	43	g/L
ALT	10	U/L
AST	15	U/L
ALP	257	U/L
Total bilirubin	18.1	mg/dL
Direct bilirubin	13.2	mg/dL
PT	12.3	s
PTT	25.5	s
INR	0.9	
BUN	4.1	mg/dL
Creatinine, serum	25.0	
Ferritin levels	2,921	mcg/dL
Transferrin saturation	295	mcg/dL

## Discussion

Hereditary hemochromatosis is a rare autosomal recessive inherited disease-causing iron overload and iron deposition in several organs, resulting in a variety of clinical presentations. In this paper, we reported a unique case of HH associated with idiopathic refractory aplastic anemia. Secondary hemochromatosis is an important differential of HH, which is usually originated by repeated blood transfusion and ineffective erythropoiesis. Thus, it is not an unusual finding in subjects with aplastic anemia to have iron overload due to frequent blood transfusions [7]. In our case, autosomal recessive hereditary hemochromatosis was found incidentally as we were doing further genetic tests to exclude inherited bone marrow failure syndromes as a cause of the pancytopenia.

Symptomatic hemochromatosis with iron overload can impair the immune system. The mechanism for the immune system impairment is made by enhancing the number and activity of CD8 T-cells and also increase of the CD8/CD4 ratio T-cells and weaken phagocytosis [8]. Thus, the subject with HH will be prone to infections due to his iron overload. In addition to the leukopenia, such mechanisms might exist in our patient, contributing to the recurrent emergency room (ER) visits with fever and watery diarrhea symptoms. However, we believe that our patient is completely asymptomatic as regard HH, and no specific treatment is needed as he has a normal transferrin saturation, which is considered one of the central tests in diagnosing HH. Despite the elevated ferritin level on the day of the presentation, this could be caused by acute-phase reaction and then exaggerated by the recurrent blood transfusions due to his refractory AA (Table 1).

There are two main HFE gene mutations that account for HH: p.C282Y and p.H63D [9]. In this report, the genetic test revealed an HFE pathogenic variant c.187C>G p.(His63Asp) that causes an amino acid change from histidine to aspartic acid at position 63. This variant was described as a disease-causing for HH [10,11]. Despite this variant being relatively common, it is still not adequately reported in the Kingdom of Saudi Arabia, particularly in the pediatric age group.

Previous literature has reported a variety of associated hematological conditions with HH, including autoimmune hemolytic anemia, congenital dyserythropoietic anemia, and red cell aplasia [12-14]. After a thorough review of the scientific literature in English, the co-existence of AA with HH has been documented only in one adult patient [15]. This emphasizes the uniqueness of this paper. However, further research is needed to establish this association and physicians should remain aware until then.

Refractory (AA) is defined as a failure of response to first-line immunosuppressive therapy (IST) with anti-thymocyte globulin and cyclosporine accompanied with the persistence of severe pancytopenia six months after IST [16]. The failure of response to IST, such as in our case, may indicate that the pathophysiology of AA is not immune-mediated or due to the presence of extreme hematopoietic stem cell exhaustion. Therefore, IST is no longer considered effective. Recent literature recommends hematopoietic stem cell transplantation as a second-line treatment for refractory AA once a suitable donor is found [17]. This was employed in our case, and the donor was the patient’s father, but the transplantation was delayed by nine months because of limited centers for bone marrow transplantation in the country, and there was no available appointment. Besides these treatments, supportive care had a central role in the management of AA in view of the pancytopenia and susceptibility of life-threatening infection. We believe the supportive care significantly contributed to the positive outcome reported here.

## Conclusions

We suggest that in children with idiopathic AA, physicians should consider a genetic test for HH, especially if there was a positive family history, and normal transferrin saturation does not exclude the disease. This paper reports a unique case of HH associated with AA that did not respond to IST and underwent bone marrow transplantation since it was the only available curative option.

## References

[1] Bacon BR, Adams PC, Kowdley KV, Powell LW, Tavill AS (2011). Diagnosis and management of hemochromatosis: 2011 practice guideline by the American Association for the Study of Liver Diseases. Hepatology.

[2] Phatak PD, Bonkovsky HL, Kowdley KV (2008). Hereditary hemochromatosis: time for targeted screening. Ann Intern Med.

[3] Salgia RJ, Brown K (2015). Diagnosis and management of hereditary hemochromatosis. Clin Liver Dis.

[4] McWhorter AG, Hill SD (1991). Conservative management for a patient with aplastic anemia without use of blood products. Case report. Pediatr Dent.

[5] Oliveira LRD, Ferreira TC, Neves FDF, Meneses ACDO (2013). Aplastic anemia associated to systemic lupus erythematosus in an AIDS patient: a case report. Rev Bras Hematol Hemoter.

[6] Miano M, Dufour C (2015). The diagnosis and treatment of aplastic anemia: a review. Int J Hematol.

[7] van Bokhoven MA, van Deursen CTBM, Swinkels DW (2011). Diagnosis and management of hereditary haemochromatosis. BMJ.

[8] Walker EM Jr, Walker SM (2000). Effects of iron overload on the immune system. Ann Clin Lab Sci.

[9] Burke W, Thomson E, Khoury MJ (1998). Hereditary hemochromatosis: gene discovery and its implications for population-based screening. JAMA.

[10] Yönal O, Hatirnaz O, Akyüz F (2007). HFE gene mutation, chronic liver disease, and iron overload In Turkey. Dig Dis Sci.

[11] Guix P, Picornell A, Parera M, Galmes A, Obrador A, Ramon MM, Castro JA (2002). Distribution of HFE C282Y and H63D mutations in the Balearic Islands (NE Spain). Clin Genet.

[12] Falahatian MI, Reza Khosravi Farsani M, Hajigholami A (2019). Hereditary hemochromatosis associated with autoimmune hemolytic anemia; a case report. J Prev Epidemiol.

[13] Fargion S, Valenti L, Fracanzani AL (2000). Hereditary hemochromatosis in a patient with congenital dyserythropoietic anemia. Blood.

[14] Adams PC (1994). Hereditary hemochromatosis and red cell aplasia. Am J Hematol.

[15] Adams P, Howlett C, Xenocostas A, Chakrabarti S (2018). Sex-specific analysis post-liver transplantation in hemochromatosis with aplastic anemia and hepatocellular carcinoma. Hepatol Commun.

[16] Scheinberg P, Young NS (2012). How I treat acquired aplastic anemia. Blood.

[17] Marsh JCW, Kulasekararaj AG (2013). Management of the refractory aplastic anemia patient: what are the options?. Blood.

